# Human–Robot Interaction Strategy of Service Robot with Insufficient Capability in Self-Service Shop

**DOI:** 10.3390/biomimetics11030213

**Published:** 2026-03-16

**Authors:** Wa Gao, Tao He, Yang Ji, Yue Kan, Fusheng Zha

**Affiliations:** 1 Co-Innovation Center of Efficient Processing and Utilization of Forest Resources, Nanjing Forestry University, Nanjing 210037, China; 2 College of Furnishings and Industrial Design, Nanjing Forestry University, Nanjing 210037, China; 8231411520@njfu.edu.cn (T.H.);; 3 School of Mechanical and Power Engineering, Henan Polytechnic University, Jiaozuo 454003, China; 4 The State Key Laboratory of Robotics and System, Harbin Institute of Technology, Harbin 150001, China

**Keywords:** human–robot interaction, robot’s insufficient capability, user experience, interaction strategy

## Abstract

This paper explores the interaction strategies of service robots in self-service shops from a user experience perspective in the case of robots with insufficient capabilities. A Yanshee robot and a self-developed localization-rotation system are employed as the experimental platform. A sales return in a self-service shop is employed as the experimental scenario. Two types of robot’s insufficient capabilities, three strategies of robots’ apology and a social interaction cue imitated from a human salesperson are considered in the design of interaction strategy between human and robot in this scenario. The results show that robots’ social insufficiency leads to more negative influence on customer experiences of fluency, comprehensibility, impression, intelligence, willingness for future interaction than robots’ performance insufficiency. An empathetic apology when the robot has insufficient performance is an effective interaction strategy. The interaction cue that the robot turns to face customers is not beneficial to customer experiences but does influence the internal relationship between customer experiences during HRI and after HRI. In the case of robots with social insufficiency in a self-service shop, impression, intelligence and interaction capability have positive impacts on the willingness for future interaction, while they are also positively affected by fluency or comprehensibility. In the case of robots with performance insufficiency, impression has a positive impact on willingness, while it is not directly related to fluency. The findings are valuable for informing the interaction design of service robots deployed in shopping, especially in real environments where performance and cost must be balanced.

## 1. Introduction

With rapid development of technologies such as artificial intelligence, machine learning, etc., service robots have been widely used in fields like retail and so on [1]. Shops represent pivotal environments within retail services. Currently, service robots deployed in retail environments have progressively acquired a range of capabilities such as greetings [2,3,4], flyer distribution [5], and consumer guidance during shopping processes [6], etc. They are expected to take on the job responsibilities that are currently carried out by human employees in the shops. Hence, researchers explore human–robot interaction (HRI) in retail environments from multiple perspectives, including the roles of service robots in shopping malls [7], interaction cues exhibited by service robots [8,9], the impact of service robots on customer shopping behaviors [10], and customer experiences interacting with service robots in the shopping process [11], and so on.

Despite the continuous evolution of service robots in recent years, they cannot always interact smoothly with humans due to the complexity of real-world scenarios and the diverse requirements of users. Some studies have mentioned that robots have insufficient abilities when facing the unstructured environments or complex instructions [12,13,14]. Some studies have also pointed out the interaction errors that make it hard for users to achieve their intention [15,16]. From the user perspective, these indicate that the service robots sometimes are unable to effectively perform the assigned tasks. The insufficient capability of service robots may affect multiple experiences of HRI for customers, including interaction fluency, comprehensibility, impressions of the robots, and willingness to use in future interactions, etc. Yam et al. have suggested that perceived experience interacts with robot service failures, which can predict customer attitudes, helping mitigate the negative effects of service failures [17]. However, there are relatively few studies on customer experience for HRI in the context of service robots’ insufficient capabilities.

Service robots are required to interact with customers and deliver services in retail environments [18]. They are required to fulfill assigned tasks such as greeting customers, providing guidance, facilitating sales, conducting patrols, etc. They also need to adhere to social norms and conform to corresponding behavioral patterns when interacting with humans. The completion of assigned tasks primarily depends on the technical capabilities incorporated during the service robot’s development, while adherence to social norms is regarded as a manifestation of the design of robots’ social strategy. For example, service robots with guiding function must be capable of detecting customer arrival. Meanwhile, they need to attract users by demonstrating social usability, thereby encouraging customers with needs to approach the service robot and engage in interaction [19,20]. Service robots with mobility functions need to perceive environment characteristics. Meanwhile, they also need to convey their own movement intentions to pedestrians through social cues, which can make their movements more readable and positively influence the acceptance and successful deployment of service robots [21]. Hence, when service robots exhibit insufficient capabilities, they can be categorized into two types: performance incapacity from the view of functions or technology and social incapacity from the view of social norms.

This study explores the HRI strategies of service robots with insufficient capacity in a self-service shop by studying the customer experiences. We focus on six aspects of customer experiences: fluency, comprehensibility, overall impression, perceived intelligence, interaction capability, and willingness for future interaction. The first research question (RQ) is as follows.

RQ1: What is the impact of the type of robot insufficient capabilities on the aforementioned aspects of customer experience in a self-service shop?

When a robot makes mistakes or shows performance failures, robot apology is recognized as an effective interaction strategy for this approach that can help repair the trust between human and robot [22,23,24]. The expression design for robot apologies incorporates human-inspired modalities, including bowing, verbal utterances, etc. [25,26,27]. Considering that apology behavior conforms to social norms, the second RQ is as follows.

RQ2: When the service robot shows performance insufficiency in a self-service shop, how can a robot apology strategy be designed through interaction cues to obtain relative better aforementioned customer experiences?

When service robots provide services to customers, they can also convey their attention through the interaction cues such as gaze, voice, body movements, etc. [28]. In particular, eye contact can influence users’ psychological and physiological responses related to emotions and attention, indicate the robot’s behavioral intentions to users, and improve their willingness to engage in interaction with the robot [21,29,30,31]. However, it should be noted that the development of service robots’ functions and the design and implementation of social strategies need to consider the constraints of their physical architectures. The incorporation of additional interactive cues into the design of social strategies entails relatively higher development costs for service robots. It means exploring simple social strategies is necessary. Considering that eye contact requires the service robot to face the users and a human salesperson or shopkeeper also needs to turn and face customers when providing service in real environments, the third RQ is as follows.

RQ3: When a service robot turns to face the customer while providing services, can this behavior serve as an effective social interaction cue in the case of robots with insufficient capabilities in a self-service shop?

Moreover, for the customer experiences of HRI, fluency refers to a high level of coordination and adaptation between human and robots [32]. The comprehensibility includes the understanding of various interaction cues during the interaction process. Both of these are experienced during the process of HRI. Overall impression, perceived intelligence, interaction capability and willingness for future interaction are the feelings of customers after the interactions. For the robots with insufficient capabilities in shopping scenarios, ensuring customers’ willingness to use them in the future constitutes an important consideration. Hence, when service robots have insufficient capabilities, the fourth and the fifth research questions are as follows.

RQ4: For customer experiences in a self-service shop, what impact does fluency and comprehensibility during the HRI have on overall impression, perceived intelligence, interaction capability and willingness for future interaction?

RQ5: For customer experiences in a self-service shop, what impact does overall impression, perceived intelligence, and interaction capability have on willingness for future interaction?

The remainder of this paper is organized as follows. Section 2 describes the related works. Section 3 describes the experiments, the design of interaction strategies, hypotheses, etc. Specifically, we describe the experiments to evaluate the customer experiences. Section 4 analyzes the data obtained from the experiments by statistical methods. Section 5 describe the discussions of the five RQs. Section 6 provides the conclusion of this paper.

## 2. Related Works

### 2.1. Service Robot in Shop

Service robots deployed in shopping environments typically perform tasks including welcoming, informing, assisting, entertaining, advertising, guiding, etc. [1,7]. Their application can not only provide customers with novelty, convenience and safety [33] but also influence their willingness to interact with robots [34,35]. When fulfilling the duties of a human salesperson, service robots can complete specific tasks through technologies such as sensors, computer vision, speech recognition, navigation systems, machine learning, etc. [36]. And they also establish a relationship with customers by interaction strategies, which are conveyed through various interaction cues [27]. For example, when service robots are engaged in guiding tasks, speech and gaze, human-like gestures, starting the interaction followed by a friendly greeting, and confirming the required location are advantageous interaction cues [37]. When improving the interactions between humans and robots on pedestrian ways, design of robot gaze cues is a feasible approach [38]. When robots are engaged in indirect handovers, body gestures, gaze or speech, and arm gestures are employed according to interaction phases to achieve polite and warm handover [39]. When providing a friendly and admonishing service for a shopworker robot, the robot is designed to roam the shop, show a friendly behavior like giving directions with voice or an admonishing behavior based on the detection of a customer’s inappropriate actions [40]. Overall, the performance capabilities and the social capabilities of service robots jointly enhance the customer experience in shopping environments. To illustrate the diverse capabilities of robots in shopping, some studies are summarized in Table 1. The above review also indicates that, although existing research has extensively explored the diverse functions of service robots in retail environments, the achievement of their current capabilities still relies on targeted design, and the behaviors of robot are unlikely to meet all the requirements of various shopping scenarios. Hence, the impact of a robot’s performance and social insufficiency requires further study.

### 2.2. Types of Insufficient Capacity for Robot

It is highly improbable for service robots to achieve full autonomy and completely eliminate errors in HRI [41,42,43]. The interaction errors in HRI are categorized into two distinct classes as performance errors and social errors from the user-centered perspective [44,45]. The performance errors are errors that reduce users’ perception of the robot’s intelligence and competence for achieving tasks [44]. They mainly focus on the functional dimension of HRI. For instance, the burger-flipping robot Flippy was taken offline for being too slow, and the grocery store robot Fabio was deemed a failure for it was unable to fully understand customers’ questions [46]. The social errors are errors that violate social norms such as interrupting customers at an inappropriate time, inappropriate expression of emotions, etc. [44]. They mainly consider the social-affective dimension of HRI. The violation of social norms by robots can elicit strong emotional reactions in users. For example, a virtual assistant robot Churi employed by the Henn-Na Hotel was “fired” for its malfunctions because it interrupted guests’ conversations and messed up room service orders [47]. There are both performance insufficiencies and social errors when it is working. Moreover, consumer perceptions to service robots depend not only on their functions but also on their social skills [48]. Hence, this paper explores the impact on customer experience from robots’ performance insufficiency and social insufficiency.

### 2.3. Robot Apology in HRI

Robot apology is a commonly employed strategy in HRI design to address robot failure, repair trust and recover HRI [26]. For example, Uchida et al. proposed a strategy for recovering from dialogue breakdown in HRI by the design of robot apology without reducing users’ motivation [49]. Zhang et al. analyzed the impacts of internal attribution apology (e.g., “Sorry, I was too timid to ask questions”) and external attribution apology (e.g., “Sorry, the question was phrased too ambiguously”) on users in HRI [22]. They found that internal attribution apology is the optimal repair strategy for the logic performance failure. Kim et al. found that internal attribution apology restores more trust than external attribution apology for competence-based violations [50]. An internal attribution apology refers to assuming full responsibilities and attributing blame to internal factors. When using internal attribution apology in HRI design, the robot should take full responsibility. Hence, exploring the ways of apology in the cases of robot performance and social insufficiency is a valuable research endeavor.

### 2.4. User Experience in HRI

When service robots are integrated into real social environments, fluency and comprehensibility are two critical aspects of user experience in HRI. Fluency can lead to positive emotional cognition [51] and also contribute to users’ perception of the intelligence and reliability of the robot [52]. The comprehensibility for service robots can help improve the interaction between humans and robots. Users can infer the robot through interaction cues such as its voice, body movements, etc. For example, Karhu et al. studied the characteristics, including robot’s speech speed, volume, etc., that make robot speech understandable to seniors for improving senior–robot interactions [53]. The study of Lu et al. mentioned that the socialization of robots should follow the principle of comprehensibility, social compliance, etc. [54].

Following interaction with service robots, certain user experiences may also influence customer attitudes of service robots. For example, Anastasiou et al. made the users’ overall impression of the robot as one of the questionnaires in the evaluations [55]. Customers’ perception of service robot intelligence is also meaningful. Robots being perceived as intelligent positively affects rapport building between customers and robots [56] and also directly and positively affects customers’ intention to continue using robots [57]. Higher levels of thinking AI and feeling AI in service robots trigger positive emotions in customers [58]. It indicates that the perception of service robot intelligence is necessary for studying the user experience in cases where the robot’s capabilities are insufficient.

Moreover, customers’ ability to interact with service robots is need to consider. Individuals’ estimates about their own abilities to perform in a particular situation or task is referred as self-efficacy [59]. It has been verified to have a direct influence on individuals’ willingness to adopt various technologies across different contexts such as online shopping, computer usage, etc. [60,61], and also represent a stronger predictor of behavioral intention for computing system tasks [62]. Therefore, the customers’ assessments of their own ability to interact with the service robot may hold predictive significance for the willingness to use the robot. The willingness to use the robot reflects the customers’ tendency to have interaction with the robot in the future. It has been studied from multiple perspectives, including interaction quality perception, interaction cues perception, robot’s social attributes, etc. [33,63,64]. The aforementioned research provides valuable insights for our study in examining customers’ experiences in self-service shop when the capabilities of service robots are insufficient.

## 3. Design and Experiments

### 3.1. The Scenario of After-Sales Service in Self-Service Shop

After-sales service is an essential component of shop operations. When service robots are used in a self-service shop, they are served as shop assistants and should also be capable of after-sales service. However, there are few studies and applications of robots in this field. We designed this scenario to explore the interaction strategies in the case of insufficiency capability of robots in a self-service shop. We have summarized the process of sales return by observation from traditional shops into three phases named as the start phase (SP), the inquiry phase (IP) and the response phase (RP), respectively.

SP: The shop assistants greet the customers upon their arrival by speaking or waving hands.

IP: The shop assistants inquire about the customer’s requirements. And then the customer describes the requirement of sales return and the relevant reasons.

RP: The shop assistants respond to the customer’s requirement of sales return, explaining whether it is possible to return the product and explaining the relevant reasons for this sales return. In this phase, we observed that some shop assistants used some emotional behaviors, expressing apology, regret or sadness to try to convey a sense of empathy for better communications. It is consistent with the studies of Refs. [65,66] that empathic behavior of service employees significantly develops satisfaction during service interactions.

### 3.2. The Performance of a Robot’s Insufficient Capabilities

According to Section 2.2, we categorized robots’ insufficient capabilities into two types, social insufficiency and performance insufficiency.

Social insufficiency is the insufficiency where robots violate social norms, which make it hard for them to fulfill users’ expected requirements. For example, when customers requested sales return, the robot provided an irrelevant response to them.

Performance insufficiency is the insufficiency where robots lack certain functionality or certain tasks fall outside the functional capabilities of the robots. For example, when customers requested sales return, the robot had not been designed to support this functionality.

### 3.3. The Experimental Platform

**Figure 1:**
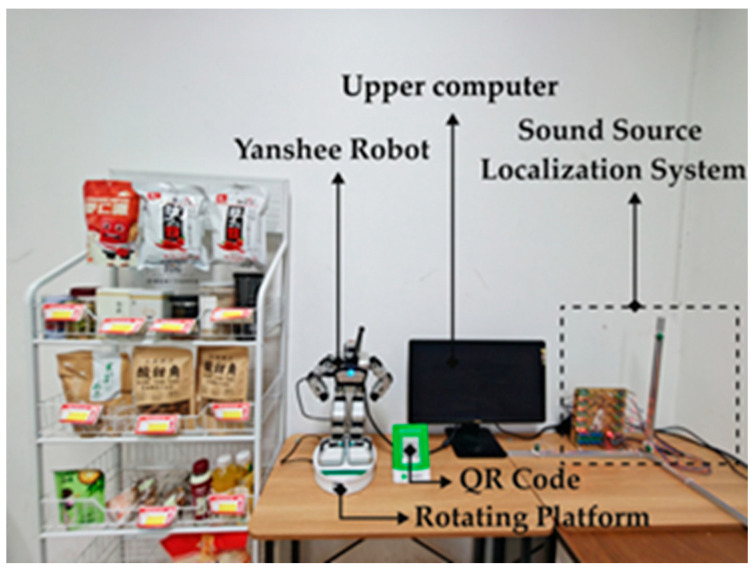
The experimental platform in self-service shopping scenario.

The experimental platform includes a humanoid robot named Yanshee, a sound source localization system, and a rotating system, as shown in Figure 1. The Yanshee robot, which has 17 degrees of freedom, can achieve interaction with humans by multiple interaction cues like voice, body language, etc. When a participant speaks to Yanshee robot, the sound source localization system can collect the voice, send the information to the computer, and the computer sends information to make the rotating system turn to face the participant. Then Yanshee robot speaks or shows body language to the participant. The scenario includes QR code payment, snack shelves, tables and chairs, etc., as shown in Figure 1, to simulate a real self-service shop.

The sound source localization system is self-developed. The hardware incorporates an optimal spatial tetrahedral microphone array, an audio conditioning circuitry, an audio acquisition module and an upper computer. The optimal spatial tetrahedral microphone array is designed to ensure the development requirements of the accuracy of azimuth angles and real-time performance. It captures the audio signals, and the audio conditioning circuitry improves the quality of the captured audio signals, and then the audio acquisition module transmits the signals to the upper computer. The azimuth angle, the pitch angle and the localization distance of sound are obtained by the Time Difference of Arrival (TDOA) and the Generalized Cross-Correlation with Phase Transformation (GCC-PHAT) algorithms. The upper computer encodes the sound source localization and then transmits it to the Yanshee robot. The robot decodes and invokes the API Interaction Content File to express different behaviors.

**Figure 2:**
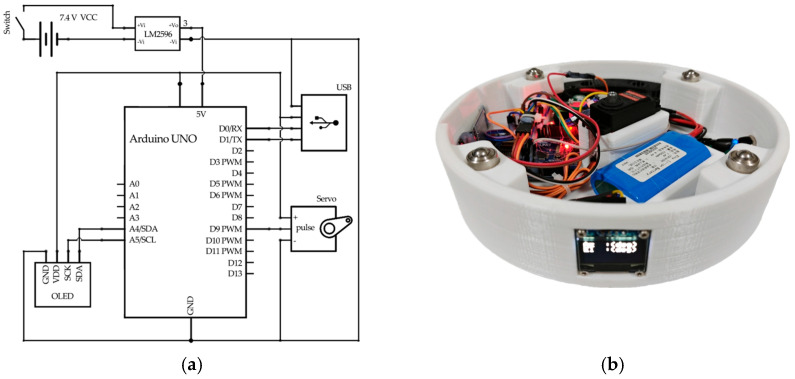
(**a**) Circuit of the rotating system; (**b**) structure of the rotating system.

The rotating system comprises an OLED display screen, a servo motor, an Arduino Uno R3 controller, power supply, etc. The corresponding circuit is shown in Figure 2a, and the structure is shown in Figure 2b. Its shell is fabricated using 3D printing technology. The Yanshee robot can send the position of localized sound source to the Arduino Uno R3 controller of the rotating system. Then the azimuth angle, the pitch angle and the localization distance of sound are displayed on the OLED screen. The servo of the rotating system rotates based on the obtained azimuth angle in order to face the users.

### 3.4. Interaction Strategies Design of Robot

The interaction behavior of the Yanshee robot when handling sales returns in the self-service shop is designed to mimic that of a salesperson based on the analysis in Section 3.1. In the start phase (SP), the Yanshee robot initiates communication with the customer and conveys a welcoming message. In the inquiry phase (IP), the Yanshee robot asks the customer about the services they need. In the response phase (RP), the responses of Yanshee robot to the customer for sale return are categorized into two types according to the two different insufficient capabilities of robots mentioned in Section 3.2.

**Figure 3:**
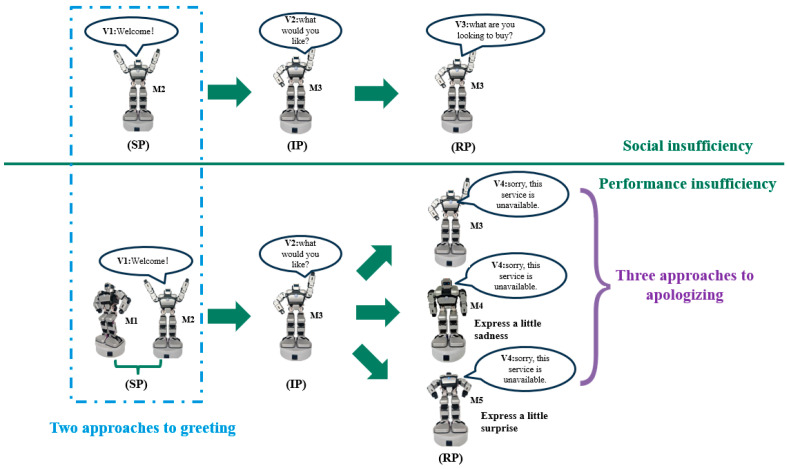
The design of robot’s voice and body movements for interaction strategies.

For the three phases, we designed the voices and the body movements for the Yanshee robot as shown in Figure 3. The robot’s voices, V1~V4, and robot’s body movements, M1~M5, are shown in Figure 3. One voice response, V3, exemplified in Figure 3 as “what are you looking to buy?”, delivers irrelevant information, which means a violation of social norms. The other voice response, V4, is “sorry, this service is unavailable” to suggest that this function is limited and assigns responsibility to the robot itself. It is an apology based on internal attribution. The movement M1 imitates the behavior where the salesperson turns to face customers, while M2 and M3 imitate the greeting and the guidance of a salesperson as shown in Ref. [67]. The body movements, M4 and M5, express different emotions based on the robot’s body movements in Ref. [68]. M4 is employed to deliver empathy when the Yanshee robot apologized in voice, while M5 is used to convey surprise at encountering something beyond the robot’s capabilities.

Totally, we consider two types of the robot’s insufficient capabilities, three approaches to apologizing, and two approaches to greeting. The interaction strategies of the corresponding eight cases are shown in Table 2. The eight cases are named from S1~S4 and SR1~SR4, respectively. In the cases of SR1~SR4, the Yanshee robot actively turns to face to the customer when it captures the customer’s voice, while, in the cases of S1~S4, the Yanshee robot does not. For the performance insufficiency, a total of three distinct apology methods, including just by voice, by voice with the body movement of a little sadness and by voice with the body movement of a little surprise, were developed.

### 3.5. Experimental Procedure

**Figure 4:**
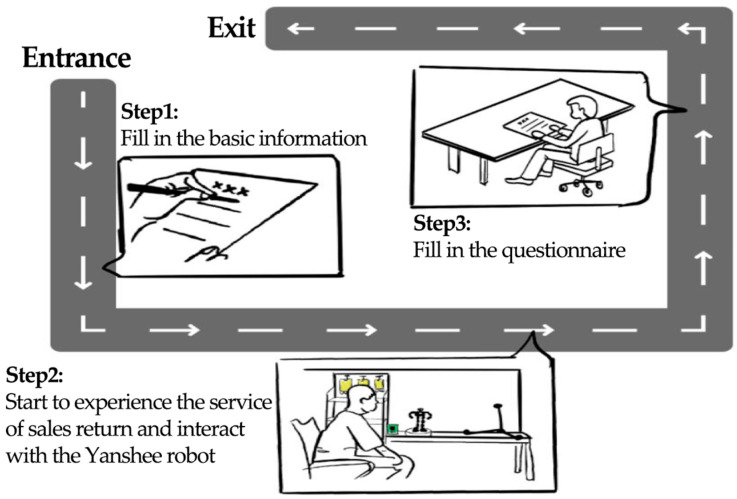
Detailed experimental steps.

We use G*Power 3.1 to determine that the minimum of participants is 16. Then 25 participants were recruited in a university campus randomly, of which 12 were males and 13 females with M = 23.24, SD = 1.77. Before the experiments, all participants were informed of the study details, and all of them provided written informed consent forms for their participation. Then they were asked to interact with the robot to experience the service of sales return. The experiments were divided into two stages, as shown in Figure 4. For the first stage, each participant experienced the cases of S1~S4 in a random order. Following each case, a short break was provided. After a few days, they experienced the cases of SR1~SR4 randomly in a random order, and a short break was also provided following each case. The two stages were separated by a sufficient interval to minimize potential carryover effects, such as learning or fatigue, and to ensure that participants’ evaluations of each condition were as independent as possible. After each case in the two steps, they were asked to complete the questionnaires as shown in Table 3. The attitudes of participants consisting of fluency, comprehensibility, impression, intelligence, interaction capability, willingness for future interaction were investigated in the questionnaire. An 11-point Likert scale, in which 11 corresponded to “strong” and 1 corresponded to “weak”, was used here.

### 3.6. Hypotheses

Considering the three RQs mentioned in Section 1 and the related work mentioned in Section 2, this study proposes the following three hypotheses:

**Hypothesis** **1 (H1).** *For the type of insufficiency in the robot’s capabilities, considering that the interaction capability refers to the customers’ own beliefs, H1a and H1b are considered, respectively.*

**H1a.**  *Compared with robot’s performance insufficiency, the robot’s social insufficiency leads to more negative influence on the customer experiences for fluency, comprehensibility, impression, intelligence, and willingness for future interaction in a self-service shop.*

**H1b.**  *The robot’s performance insufficiency and robot’s social insufficiency do not significantly influence the customer’s interaction capability in a self-service shop.*

**Hypothesis** **2 (H2).** *When robots have performance insufficiencies, for the design of service robots’ apology, compared with robots’ verbal apologies and robots’ verbal apologies with body movements of surprise, two aspects are considered.*

**H2a.**  *Robots’ verbal apologies with body movements expressing empathy can positively influence on the fluency, comprehensibility, impression, intelligence, and willingness for future interaction in a self-service shop.*

**H2b.**  *Robots’ verbal apologies with body movements expressing empathy do not significantly influence the customer’s interaction capability in a self-service shop.*

**Hypothesis** **3 (H3).** *When robots have insufficient capabilities, compared with robots that do not face the customers, robots that can turn to face the customers can improve the fluency, comprehensibility, impression, intelligence, interaction capability, willingness for future interaction in a self-service shop.*

## 4. Results and Analysis

The data obtained by the questionnaires were analyzed by the statistical methods. The mean values (M-values) and the standard deviations (SDs) of the scores for the six aspects of attitudes are shown in Table 4. They are approximately a normal distribution.

### 4.1. Reliability and Validity of Questionnaire

The Cronbach’s α reliability coefficients and Kaiser–Meyer–Olkin (KMO) values for the eight experimental scenarios are presented in Table 5. All Cronbach’s α values exceed 0.70, indicating good internal consistency of the questionnaire items. The KMO values range from 0.624 to 0.832, with most above 0.60, suggesting that the data are suitable for factor analysis. Additionally, Bartlett’s Test of Sphericity yielded significance levels below 0.001 for all scenarios, confirming that the correlation matrix is not an identity matrix and that factor analysis is appropriate. These results collectively demonstrate that the questionnaire data are reliable and valid for measuring the six dimensions of customer experience.

### 4.2. The Results of Repeated-Measures ANOVA

**Figure 5:**
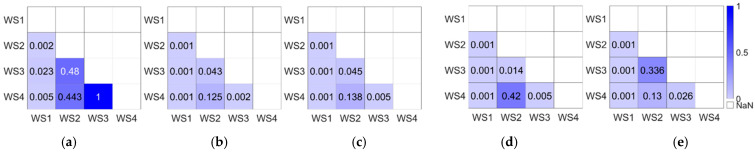
Heatmaps of pairwise comparison *p*-values for within-subjects effects: (**a**) fluency, (**b**) comprehensibility, (**c**) impression, (**d**) intelligence, and (**e**) willingness for future interaction. Each cell represents the *p*-value of the comparison between two conditions (WS1 to WS4). Color intensity indicates the significance level: lighter colors correspond to smaller *p*-values (more significant differences).

According to whether the Yanshee robot turned to face the participant, the data were divided into two groups to analyze. One group is the data obtained from S1~S4, in which the Yanshee robot did not turn to face the participant, while another group is the data obtained from SR1~SR4. The repeated-measures ANOVA is used. Considering the *p* values of the Mauchly’s test of sphericity, the results of the tests of between-subjects effects and the tests of within-subjects effects are shown in Table 6. All *p* values of the tests of between-subjects effects are larger than 0.05. This indicates that there are no significant differences whether the Yanshee robot turned to face the participant or not for the six factors. For the results of within-subjects effects, the pairwise comparisons were implemented for the attitudes of fluency, comprehensibility, impression, intelligence, and willingness for future interaction, since the corresponding *p* values of the tests of within-subjects effects is smaller than 0.05. The results are shown in Figure 5, where WS1 represents the group of social insufficiency and WS2~WS4 represent the groups of performance insufficiency with different apology design. The corresponding apology methods are shown by the response phases in Figure 3. For WS2, the robot apologizes by voice. For WS3, the robot apologizes by voice accompanied with body movement expressing a little sadness. For WS4, the robot apologizes by voice accompanied with body movement expressing a little surprise.

## 5. Discussion

### 5.1. The Impact of Different Robot’s Insufficient Capabilities on Customer Experiences

H1a is supported. Compare WS1 with WS2, WS3, and WS4 based on Figure 5 and Table 4. For the customer experiences of fluency, comprehensibility, impression, intelligence, and willingness for future interaction, the scores for social insufficiencies (WS1) are significantly lower than those for performance insufficiencies (WS2, WS3, and WS4) across all apology types.

H1b is supported. For the customer’s interaction capability, there are no significant differences between the scores of social insufficiencies (WS1) and performance insufficiencies (WS2, WS3 and WS4).

Hence, for RQ1, compared with the robot’s performance insufficiency, the robot’s social insufficiency leads to more negative influence on the fluency, comprehensibility, impression, intelligence, and willingness for future interaction in a self-service shop with robot assistants. The types of the robot’s insufficient capabilities have no influence on the customer’s interaction capability.

### 5.2. The Impact of Robot’s Apology Design on Customer Experiences in the Case of Performance Insufficiency

H2a is partially supported. Compare WS3 with WS2 and WS4 based on Figure 5 and Table 4. For the customer experiences of comprehensibility, impression and intelligence, the scores of verbal apologies accompanied with body movements of sadness (WS3) are significantly higher than those of verbal apologies (WS2) and verbal apologies accompanied with body movements of surprise (WS4). For willingness for future interaction, the scores of verbal apologies accompanied with body movements of sadness (WS3) are significantly higher than those of verbal apologies accompanied with that of body movements of surprise (WS4) but do not show significantly difference with those of verbal apologies (WS2).

Moreover, for fluency and interaction capability, there are no significant differences between the three types of apologies in the case of the robot’s performance insufficiency. This indicates that H2b is supported. In addition, for the mentioned six aspects of customers’ experiences, the scores of WS2 and WS4, there are no significant differences. This indicates that the surprise body movement of the robot has no significant effect and may reduce the customer’s willingness to use for future interaction in the case of the robot’s performance insufficiency. Hence, for RQ2, the robot’s body movements expressing sadness can lead to higher customer ratings of comprehensibility, impression, intelligence, and willingness for future interaction. This is to some extent similar to the role of salesperson’s empathy, as mentioned in Ref. [66], where employees with empathy are more likely to gain customer trust, enhance customer satisfaction, and ensure smooth future interactions.

### 5.3. The Impact of Robot Turning to Face to Customers in the Cases of Robot’s Insufficient Capabilities

H3 is not supported. According to the data in Table 6, there are no significant differences, since all *p* values of the tests of between-subjects effects are larger than 0.05. This indicates that, for RQ3, in the cases of the robot’s insufficient capabilities, whether the robot turns to face the customers to provide services has no influence on customer attitudes of fluency, comprehensibility, impression, intelligence, interaction capability and willingness for future interaction in a self-service shop with robot assistants.

### 5.4. The Internal Relations Among the Six Customer Experiences in the Cases of Robot’s Insufficient Capabilities

The internal relations among the six customer experiences are discussed from the perspectives of the robot’s social insufficiency and the robot’s performance insufficiency. As shown in Table 2, the cases, S1 and SR1, are those of a robot with social insufficiency. S3 and SR3 are the cases of a robot with performance insufficiency obtaining the best evaluations of customer experiences in this paper. Even though the robot’s performances are insufficient in S3 and SR3, the robot’s feedback to customers is designed to be generally consistent with social norms. In addition, turning to face customers is an approach more suitable to social norms. It means that, compared with S1 and S3, the social norm of SR1 and SR3 in their own cases are relatively better. The design details of interaction strategies of the four cases are shown in Section 3.4. Hence, the data obtained by these four cases are analyzed to explore RQ4 and RQ5.

#### 5.4.1. The Results of Regression Analysis

The correlations of fluency, comprehensibility, impression, intelligence, interaction capability and willingness for future interaction in the cases of S1, SR1, S3 and SR3 are analyzed by Spearman correlation test. The results show that there are correlations among the six aspects of customer experiences in the case of S1, with all *p* values being smaller than 0.05. In the case of SR1, fluency has no correlation with interaction capability (*p* = 0.362), while others show correlations. In the case of S3, impression has no correlation with interaction capability (*p* = 0.081), and willingness for future interaction has no correlations with intelligence and interaction capability (*p* = 0.08 and *p* = 0.17, respectively). In the case of SR3, fluency has no correlation with impression (*p* = 0.068), comprehensibility has no correlation with impression, intelligence and willingness for future interaction (*p* = 0.247, *p* = 0.117 and *p* = 0.051, respectively), and intelligence also has no correlation with interaction capability (*p* = 0.08).

Based on the above correlation analysis, the multivariate regression analysis is employed to explore RQ4. The fluency and comprehensibility are used as independent variables. Impression, intelligence, interaction capability and willingness for future interaction are used as dependent variables. It means analyzing the impact of the customer experiences during the HRI on the feelings after the HRI under this scenario. The corresponding results where *p* values of both the ANOVA and the regression analysis coefficients are smaller than 0.05 are shown in Table 7. The simple regression analysis is employed to explore RQ5. The results where *p* values of both the ANOVA and the regression analysis coefficients are smaller than 0.05 are shown in Table 8 and Table 9. All the corresponding VIF of these regression analyses are smaller than 5.

#### 5.4.2. The Models of Internal Relations Among the Customer Experiences in the Case of Robot with Insufficient Capabilities

**Figure 6:**
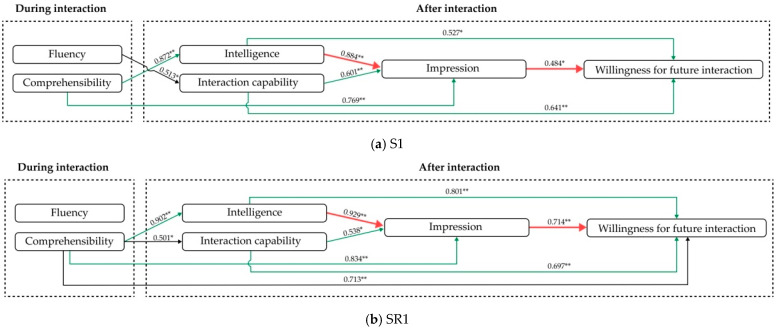
The models describing positive influence in the case of service robot with social insufficiency. Numbers represent standardized regression coefficients (Beta). Significance levels: * *p* < 0.05, ** *p* < 0.01.

The cases where service robots have social insufficiencies are considered first. According to Table 7, in the case of S1, comprehensibility shows a positive impact on impression, while intelligence and fluency show a positive impact on interaction capability. In the case of SR1, comprehensibility shows a positive impact on impression, intelligence, interaction capability and willingness for future interaction. According to Table 8, both intelligence and interaction capability show positive impacts on impression in the cases of S1 and SR1. According to Table 9, intelligence, interaction capability and impression show positive impacts on willingness for future interaction. Hence, the models describing positive influence in the case of a robot with social insufficiency are summarized in Figure 6a,b.

**Figure 7:**
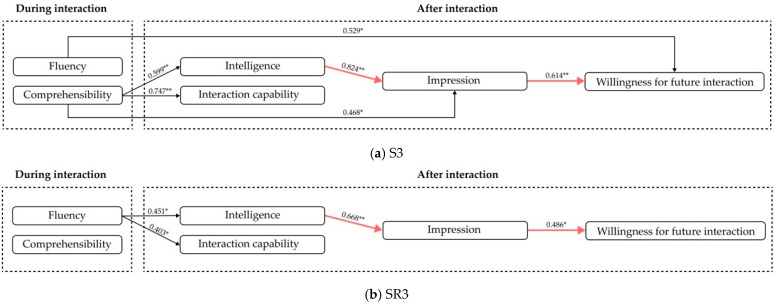
The models describing positive influence in the case of robot with performance insufficiency. Numbers represent standardized regression coefficients (Beta). Significance levels: * *p* < 0.05, ** *p* < 0.01.

Then, for the cases of service robots with performance insufficiencies, according to Table 7, comprehensibility shows positive impacts on intelligence, interaction capability and impression in S3, while fluency has direct positive impacts on willingness for future interaction. According to Table 8 and Table 9, intelligence shows direct positive impacts on impression and thereby shows impacts on willingness for future interaction. Hence, the models describing positive influences in the case of a robot with performance insufficiency are summarized in Figure 7a,b.

#### 5.4.3. The Discussions of Internal Relations Among the Customer Experiences in the Case of Robot with Insufficient Capabilities

For RQ4, when service robots have social insufficiencies, comprehensibility shows a direct positive impact on intelligence and impression. When the social norm is relatively lower (S1), fluency directly influences the customer’s interaction capability, thereby affecting their willingness to interact, and comprehensibility directly influences the impression, thereby affecting their willingness to interact. When the social norm is relatively higher (SR1), fluency no longer has a significant effect; instead, comprehension directly and positively affects their willingness to interact in future. The black arrows denote the differences in the influence relationship between Figure 6a,b. 

For RQ4, in the cases where robots have insufficient performances, when the social norm is relatively lower (S3), fluency can directly influence the willingness, while comprehensibility shows a positive impact on impression and thereby influences the willingness. When the social norm is relatively higher (SR3), although fluency has a positive impact on intelligence and interaction capability, it has no direct relationship with the willingness for future interaction.

For RQ5, according to the red arrows shown in Figure 6 and Figure 7, the similarities when service robots have social insufficiency and performance insufficiency can be seen. That is, the intelligence shows direct positive impacts on impression and thereby shows impacts on willingness for future interaction. The green arrows in Figure 6 are the similar supplements to the finding that interaction capability and intelligence can also directly positively affect willingness for future interaction in the case of a robot with social insufficiencies (S1 and SR1).

The findings indicate that impression shows as a mediating factor to positively affect the willingness for future interaction when robots have insufficient capabilities. This implies that interaction strategies can be developed from the perspective of enhancing customer impressions, thereby increasing their willingness in future interactions. In the case of social insufficiency, improving comprehensibility and intelligence are direct and effective approaches to positively affect the willingness for future interaction, while, in the case of performance insufficiency, improving intelligence does.

## 6. Conclusions

This paper primarily examined six aspects of customer experience, including fluency, comprehensibility, overall impression, perceived intelligence, interaction capability, and willingness for future interaction, to explore the interaction strategies under conditions of robots’ insufficient capabilities. We built a robotic system as the experimental platform consisting of a humanoid robot named Yanshee, a sound source localization system, and a rotating system and established a self-service shopping scenario for the implementation of the experiments. We divided the types of robots’ insufficient capabilities into social insufficiency and performance insufficiency and designed the robot apologies through three approaches, verbal, verbal with body movement expressing empathy, and verbal with body movement expressing surprise. Totally, eight interaction strategies are designed to explore the effective strategy for the scenario of a robot’s insufficient capabilities. The contributions of this paper are as follows:

1. The impacts of the robot’s insufficiency on the mentioned six aspects of customer experiences are discussed. The robot’s social insufficiency shows more negative influence on fluency, comprehensibility, impression, intelligence, and willingness for future interaction compared with the robot’s performance insufficiency.
2. The impacts of robot apology with empathy on the mentioned six aspects of customer experiences and the comparisons with verbal apology and verbal apology with surprised body movement are discussed. The robot’s body movements expressing empathy can obtain better experiences of comprehensibility, impression, intelligence, and willingness for future interaction. This can provide valuable insights for interaction recovery strategies in scenarios where robotic capabilities are limited.
3. The robot’s social interaction cue where it turns to face customers does not affect the mentioned six aspects of customer experiences. However, it can influence the internal relations among these customer experiences.
4. The internal relations between the mentioned six aspects of customer experiences are discussed. With the aim of the willingness for future interaction, these relationships can help determine the appropriate design direction.

It also suggests that adherence to social norms is crucial in practical robot design; especially when balancing cost and performance or for infrequently used functions, designing interaction strategies in accordance with social norms is a feasible and effective approach to improve customer experiences.

This study has some limitations. First, the participants were limited to young adults, which restricts the generalizability of the findings to older populations. Second, the experiments were conducted in a controlled laboratory setting rather than a real self-service shop, which may limit the results being applied to real retail environments. Third, the robot’s responses were pre-scripted to ensure experimental control. While this approach was necessary for systematically comparing interaction strategies in a sales return scenario with a clear transactional goal, it does not capture the potential for more dynamic and adaptive interactions. Therefore, first, future studies will include a more age-diverse sample to examine potential generational differences in robot interaction preferences, thereby enhancing the generalizability of the findings. Second, future research will explore the integration of generative AI, such as large language models (LLMs), to optimize human–robot interaction strategies and identify more adaptive and natural interaction patterns. Finally, future studies could also incorporate objective behavioral studies related to fault diagnosis and fault tolerance, thereby complementing the results obtained in this study.

## Figures and Tables

**Table 1:** Summary of some representative studies on service robots in retail environments.

Study	Robot Type	Scenario	Key Capabilities	Main Findings
Kanda et al. (2010) [7]	Communication robot	Shopping mall	Human detection, speech interaction, guidance, etc.	Robots can attract and guide customers, but speech recognition errors reduce engagement; human operators needed to supplement capabilities.
De Gauquier et al. (2021) [20]	Entertaining robot	Retail store	Entertainment, etc.	Robot placement affects customer attention and store entry; social presence positively influences shopping behavior.
Heikkilä et al. (2019) [37]	Social robot (Pepper)	Shopping mall	Route guidance, information providing, interaction with customers, etc.	Identified nine design implications for robot guidance behavior: help start interaction, confirm asked location, give short instructions with clear structure, inform distance to destination, use gestures and landmarks appropriately, ensure equality between retails, and stay flexible.
Edirisinghe et al. (2023) [40]	Shopworker robot	Retail shop	Guidance, admonishing behavior, etc.	Customers had a positive attitude toward the proposed harmonized dual-service robot and showed a high intention to use it in the future.

**Table 2:** Eight interaction strategies in the scenario of sales return in self-service shop.

Cases	Start Phase (SP)	Inquiry Phase (IP)	Response Phase (RP)	Insufficient Capabilities
S1	M2 + V1	M3 + V2	V3	Social insufficiency
S2			V4	Performance insufficiency
S3			M4 + V4	
S4			M5 + V4	
SR1	M1 + M2 + V1		V3	Social insufficiency
SR2			V4	Performance insufficiency
SR3			M4 + V4	
SR4			M5 + V4	

**Table 3:** The descriptions of customer attitudes from six aspects.

Dimension	Experience	Details
During interaction	Fluency	How would you rate the fluency during HRI?
	Comprehensibility	To what extent do you believe the robot can comprehend your intended meaning during HRI?
After interaction	Impression	How would you rate your overall impression of HRI?
	Intelligence	To what extent do you agree that this robot demonstrates intelligence?
	Interaction capability	To what extent do you believe you possess the ability to interact with the robot?
	Willingness for future interaction	To what extent are you willing to engage in communication with the robot in the future?

**Table 4:** The M-values and the SDs of the scores for the customer experiences in eight cases.

Experiences	S1	S2	S3	S4	SR1	SR2	SR3	SR4
	M-Value (SD)							
Fluency	8.88 (1.30)	9.60 (1.00)	9.28 (1.86)	9.24 (1.39)	8.36 (2.23)	9.44 (1.87)	9.36 (1.78)	9.40 (1.58)
Comprehensibility	6.44 (2.61)	9.08 (1.41)	9.44 (1.64)	8.56 (2.10)	6.04 (2.75)	9.40 (1.44)	9.96 (1.14)	9.08 (1.55)
Impression	7.56 (2.57)	8.68 (1.73)	9.16 (1.70)	8.56 (1.85)	7.04 (2.61)	8.96 (1.86)	9.40 (1.08)	8.44 (2.02)
Intelligence	7.04 (2.54)	8.36 (1.71)	8.92 (1.91)	8.04 (2.25)	6.61 (2.53)	8.36 (2.02)	9.20 (1.16)	8.20 (1.96)
Interaction capability	9.36 (2.16)	9.88 (0.93)	9.27 (1.43)	9.64 (1.68)	9.40 (1.68)	9.76 (1.64)	10.20 (1.12)	9.56 (1.26)
Willingness for future interaction	8.16 (2.58)	9.00 (1.78)	9.12 (1.86)	8.60 (2.08)	7.16 (2.64)	8.80 (2.20)	9.08 (2.00)	8.52 (1.87)

**Table 5:** The reliability and validity of questionnaire.

Cases	S1	S2	S3	S4	SR1	SR2	SR3	SR4
Cronbach’s *α*	0.910	0.851	0.884	0.891	0.931	0.906	0.712	0.880
KMO	0.810	0.674	0.698	0.744	0.832	0.827	0.624	0.672

**Table 6:** The results of the tests of between-subjects effects and the tests of within-subjects effects by the repeated-measures ANOVA.

Experiences	Tests of Between-Subjects Effects			Tests of Within-Subjects Effects		
	*F*	*p*	*η* ^2^	*F*	*p*	*η* ^2^
Fluency	0.104	0.748	0.002	4.236	0.007	0.081
Comprehensibility	0.382	0.539	0.008	50.738	<0.001	0.514
Impression	0.005	0.944	0.000	16.466	<0.001	0.255
Intelligence	0.063	0.803	0.001	22.306	<0.001	0.317
Interaction capability	0.064	0.801	0.001	2.218	0.089	0.044
Willingness for future interaction	0.402	0.529	0.008	12.17	<0.001	0.206

**Table 7:** The results of multivariate regression analysis.

Case	Dependent Variable	Model Summary		ANOVA	Independent Variable	Coefficient	
		*R* ^2^	*D*-*W*	*F*		Standardized Coefficients Beta	*t*
S1	impression	0.767	1.534	36.193	comprehensibility	0.769	5.72
	intelligence	0.766	2.326	35.943	comprehensibility	0.872	6.466
	interaction capability	0.453	1.69	9.1	fluency	0.513	2.488
SR1	impression	0.786	1.895	40.449	comprehensibility	0.834	6.283
	intelligence	0.82	2.279	50.067	comprehensibility	0.902	7.403
	interaction capability	0.251	1.767	7.717	comprehensibility	0.501	2.778
	willingness for future interaction	0.645	2.085	20.015	comprehensibility	0.713	4.168
S3	impression	0.547	1.486	13.273	comprehensibility	0.486	2.832
	intelligence	0.548	2.258	13.355	comprehensibility	0.599	3.498
	interaction capability	0.439	1.64	8.607	comprehensibility	0.747	3.916
	willingness for future interaction	0.59	2.665	15.828	fluency	0.529	3.247
SR3	intelligence	0.203	2.004	5.869	fluency	0.451	2.423
	interaction capability	0.162	2.204	4.453	fluency	0.403	2.11

**Table 8:** The results of simple regression analysis when the dependent variable is impression.

Case	Model Summary		ANOVA	Independent Variable	Coefficient	
	*R* ^2^	*D*-*W*	*F*		Standardized Coefficients Beta	*t*
S1	0.782	1.639	82.505	intelligence	0.884	9.083
	0.362	1.371	13.034	interaction capability	0.601	3.61
SR1	0.863	1.996	144.603	intelligence	0.929	12.025
	0.289	2.482	9.353	interaction capability	0.538	3.058
S3	0.679	1.982	48.658	intelligence	0.824	6.976
SR3	0.446	1.38	18.548	intelligence	0.668	4.307

**Table 9:** The results of simple regression analysis when the dependent variable is willingness for future interaction.

Case	Model Summary		ANOVA	Independent Variable	Coefficient	
	*R* ^2^	*D*-*W*	*F*		Standardized Coefficients Beta	*t*
S1	0.234	2	7.017	impression	0.484	2.649
	0.278	1.747	8.851	intelligence	0.527	2.975
	0.411	2.062	16.051	interaction capability	0.641	4.006
SR1	0.509	2.471	23.858	impression	0.714	4.884
	0.642	2.334	41.235	intelligence	0.801	6.421
	0.486	2.578	21.781	interaction capability	0.697	4.667
S3	0.378	2.212	13.951	impression	0.614	3.735
SR3	0.237	2.081	7.13	impression	0.486	2.67

## Data Availability

The data presented in this study are available on request from the corresponding author. The data are not publicly available due to privacy.
